# Future forest aboveground carbon dynamics in the central United States: the importance of forest demographic processes

**DOI:** 10.1038/srep41821

**Published:** 2017-02-06

**Authors:** Wenchi Jin, Hong S. He, Frank R. Thompson, Wen J. Wang, Jacob S. Fraser, Stephen R. Shifley, Brice B. Hanberry, William D. Dijak

**Affiliations:** 1 School of Natural Resources, University of Missouri, 203 ABNR Building, Columbia, MO 65211, USA; 2 Northern Research Station, United States Department of Agriculture Forest Service, 202 ABNR Building, Columbia, MO 65211, USA

## Abstract

The Central Hardwood Forest (CHF) in the United States is currently a major carbon sink, there are uncertainties in how long the current carbon sink will persist and if the CHF will eventually become a carbon source. We used a multi-model ensemble to investigate aboveground carbon density of the CHF from 2010 to 2300 under current climate. Simulations were done using one representative model for each of the simple, intermediate, and complex demographic approaches (*ED2, LANDIS PRO*, and *LINKAGES*, respectively). All approaches agreed that the current carbon sink would persist at least to 2100. However, carbon dynamics after current carbon sink diminishes to zero differ for different demographic modelling approaches. Both the simple and the complex demographic approaches predicted prolonged periods of relatively stable carbon densities after 2100, with minor declines, until the end of simulations in 2300. In contrast, the intermediate demographic approach predicted the CHF would become a carbon source between 2110 and 2260, followed by another carbon sink period. The disagreement between these patterns can be partly explained by differences in the capacity of models to simulate gross growth (both birth and subsequent growth) and mortality of short-lived, relatively shade-intolerant tree species.

The Central Hardwood Forest (CHF) covers a large portion of the eastern United States and is one of the largest forested biomes in the world^1^. The CHF is currently a major carbon sink (i.e. positive net change of carbon density), similar to many other major temperate forests in the world e.g.^2^,^3^. The CHF is a carbon sink because forests are relatively young (approximately 70% of stands are between 40 and 90 years, Pan *et al*.^4^) and regrowing from historic disturbances, especially agricultural land abandonment and exploitative timber harvesting between the 1880 s and the 1940 s^5^,^6^,^7^. The CHF provides ecosystem services at regional and global scales that include carbon sequestration and critical socio-economic goods and services related to carbon dynamics^8^. Thus, the future carbon dynamics of CHF are of great interest to scientists, land owners, resource managers and policy makers.

There is no general consensus regarding the long-term future aboveground carbon dynamics of temperate forests. Three major trajectories have been proposed for the future status of these current carbon sinks based on empirical and theoretical studies: short-term, mid-term, and long-term carbon sink trajectories. The short-term carbon sink trajectory is based on field measurements of second-growth forest and suggests the forest will only remain a carbon sink for decades (approximately 80 years), and will then function as a carbon source, largely due to mortality of aging trees e.g.^9^. The mid-term carbon sink trajectory proposes the forest will remain a carbon sink longer after the forest matures until it approaches the average longevities of the current early-successional tree species. The forest will then become a carbon source due to mortality of early-successional tree species, but it will subsequently become carbon neutral as it transitions to old-growth forest dominated by late-successional tree species in an equilibrium state. This is considered a typical trajectory for even-aged forests^10^. The long-term carbon sink trajectory proposes that the aboveground carbon sink could remain for centuries (ca. 350 years) after the forest matures, however, this trajectory is inferred from studies on old-growth forests and may provide limited insight for aboveground carbon dynamics in younger forests e.g.^11^,^12^,^13^. Given that the CHF is relatively young and has a unimodal age distribution typical of even-aged forest^4^, we hypothesize that its aboveground carbon dynamics will follow the mid-term carbon sink trajectory described above and become a carbon source after approximately 150 years of regrowth when the current cohort of early-successional species reach longevity and experience an increased rate of tree mortality.

Forest aboveground carbon dynamics are influenced by intrinsic and extrinsic factors, among which forest demographic processes, play an important role e.g.^14^,^15^. Forest demographic processes consist of two major components: gross growth (including both birth and subsequent growth of trees) and mortality. Three major approaches of representing these demographic processes have been adapted by process-based forest models that are widely used to predict future aboveground carbon dynamics. A *simple demographic approach* assumes that the chance of birth is equal for all species (groups) regardless of their physiological differences. Subsequent increments in carbon (or biomass) are determined by environmental factors and physiological differences for different species (groups). Tree mortality in the model is triggered a) by longevity (all cohorts have potential to reach longevity), b) when trees are predicted to have a small or negative carbon balance, and c) when small-scale random disturbances representing density-independent stochastic weather or pest events^16^ are modelled to occur. An *intermediate demographic approach* has different chances of birth for different species or groups based on shade tolerance, seed production, and other environmental conditions. Subsequent carbon and biomass increments are based on preset growth rate curves for different species (groups). Mortality estimates are based on a combination of longevity (many cohorts have the potential to reach species’ longevity), competition among tree cohorts, and small-scale random processes^17^,^18^. In a c*omplex demographic approach*, tree birth is similar to that in the intermediate demographic approach, with subsequent tree carbon increments determined by preset growth rate curves for different species (groups) as well as by environmental factors. Mortality is triggered by longevity (only a small percentage of cohorts can reach longevity), inter-tree competition, carbon balance, and small-scale random processes^19^.

Comparison of different approaches in models and differences in model predictions can be useful^20^,^21^,^22^. If among different approaches there is a consensus on predicted future forest dynamics, then scientists, policy makers and stakeholders can be more confident in the predictions^22^. Differences among predictions can be at least partly attributed to differences in approaches and may give insight into factors driving the results^20^,^23^.

We used three process-based forest models: ED2, LANDIS PRO, and LINKAGES to simulate aboveground carbon density of the CHF from 2010 to 2300. These three widely used models were chosen based on their representativeness in simple, intermediate, and complex demographic approaches, respectively. Climate change e.g.^24^ and various large-scale disturbances e.g.^2^,^25^ have been suggested to have profound effects on forest aboveground carbon dynamics. However, most studies have not separated such effects from forest demography and conclusions drawn from such studies without a substantial understanding of effects of forest demography could be ambiguous. Therefore, we focused on understanding the effects of forest demography on aboveground carbon dynamics and conducted simulations under current climate and without major disturbances. We aimed to address the following research questions: (1) How long will current carbon sink of CHF persist? (2) After the current carbon sink is diminished to zero, would the forests of the CHF change to a carbon source? (3) How do different approaches to modelling forest demographic processes affect estimates of forest aboveground carbon dynamics?

## Methods

### Study area

**Figure 1:**
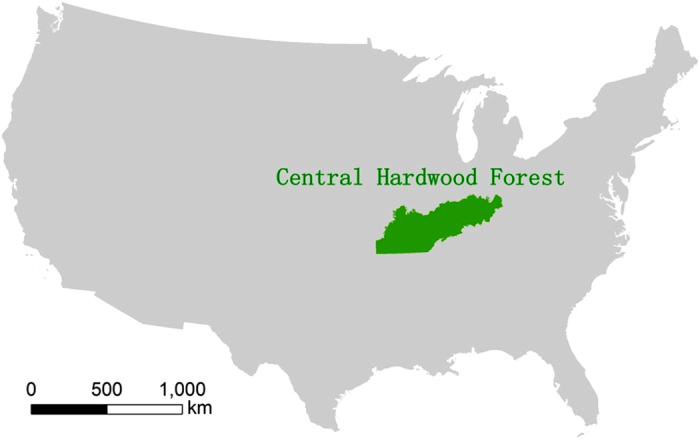
Distribution of the Central Hardwood Forest in the United States. Map was made using ArcMap 10.3 (http://desktop.arcgis.com/en/arcmap/).

The CHF covers more than 1.7 × 10^5^ km^2^ in southern Missouri, Illinois and Indiana between 36°30′ and 39°35′ N and 84°47′ and 94°36′W (Fig. 1). The climate is humid continental with long hot summers and cool winters. Mean annual temperatures range from 12.3 °C in southern Indiana to 13.1 °C in southern Missouri and mean annual precipitation ranges from 1140 mm in southern Indiana to 1090 mm in southern Illinois and 1115 mm in southern Missouri^8^. Soils in southern Missouri are moderately well drained to well-drained and are generally shallow, stony, highly weathered, and acidic. Soils in southern Illinois vary with section and topography; they include soils similar to Missouri as well as poorly drained soils such as fragipans and claypans. Soils in southern Indiana are generally well drained and typically have silt loam or loam textures^26^. The CHF region includes 53 ecological subsections in the Central Interior Broadleaf Forest ecological province^26^. About 40% of the study area is forested land, of which two-thirds is in Missouri and the remaining is divided roughly equally between Illinois and Indiana. Oak-hickory forests dominate in all three states and represent 79% of the total forest land^8^. White oak, black oak, post oak, scarlet oak, northern red oak, shagbark hickory, and pignut hickory (see Table 1 for scientific names) account for more than 75% of the total aboveground woody carbon. Shortleaf pine is the most common conifer species. The study area experienced wide-spread timber harvesting, forest clearing, and subsequent agricultural land abandonment between the 1880 s and the 1940 s^5^,^6^.

### Forest Inventory and Analysis (FIA) data

FIA is an inventory program that systematically samples, analyzes and publishes data describing forest conditions in the United States. It is managed by the United States Department of Agriculture, Forest Service. The FIA inventory is carried out on a state-by-state basis with a sampling intensity of approximately one plot per 2430 hectares^27^. Each FIA plot consists of four subplots of 7.32 m radius, in which trees with a diameter at breast height (dbh) of 12.7 centimeters or larger are measured. A microplot (2.07 m radius) is nested within each subplot, and in each microplot, trees with a dbh between 2.5 and 12.6 centimeters are measured. The FIA data include a per-hectare expansion factor for each measured tree so per-hectare estimates of forest conditions can be computed for the region or for subregions. Most FIA plots are reinventoried on cycle of approximately 10 years^27^,^28^. FIA data have been used extensively for regional forest biomass/carbon estimation and model validation e.g.^29^.

### Model calibration and validation

We used FIA data for model calibration and validation (see supplementary information). Since large-scale independent time series of spatiotemporal data for model validation rarely exist, we used a data splitting method for calibrating and validating models. Specifically, we used 50% of the FIA plots from the 1980 s to 2010 s for model calibration (calibration subset) and reserved the other 50% of FIA plots for short-term model validation (validation subset)^29^. In model validation, simulations were run from 1980 to 2010 using FIA plots from the 1980 survey cycle located within the study area. Model predictions of aboveground carbon density of (1) all plant functional types, (2) early-successional deciduous trees, (3) mid-successional deciduous trees, (4) late-successional deciduous trees, and (5) pines (see Table 1 for species in each plant functional type) in 1990, 2000 and 2010 were compared with corresponding FIA data. All predictions were averaged by ecological subsections. Standard deviations were also calculated among ecological subsections. Percent bias and bias were calculated based on the comparisons with FIA data^30^. We also compared average annual aboveground carbon density change from 1980 to 2010 predicted by three models with FIA data.

### Design of simulation

We initialized forest conditions for each model based on conditions of 2176 FIA plots that were inventoried in the CHF during the 2010 survey cycle, and from those initial conditions we ran simulations from 2010 to 2300. We recycled the observed climate conditions from 1980–2009 over the entire simulation period so climate conditions were representative of the current climate. We included 20 common tree species (Table 1), which account for more than 90% of the aboveground live carbon in the CHF. LINKAGES and LANDIS PRO modeled individual species, however, we had to assign species into 4 plant functional types for ED2 (Table 1). Replicated simulations for LINKAGES and LANDIS PRO were necessary to capture variation in outputs due to stochastic processes. We replicated simulations 20 times for LINKAGES and 5 times for LANDIS PRO. Since variation in model output for each model was extremely small, we only used one replicate for analysis. ED2 is a deterministic model, so no replications were necessary for it.

For each model applied across the entire study area we predicted total aboveground carbon density, net aboveground carbon density change, aboveground gross growth (including birth and subsequent growth), aboveground mortality. We calculated standard deviations among ecological subsections for all three models. We also reported carbon densities by species (LINKAGES, LANDIS PRO) and by plant functional type (ED2) for the entire study area.

## Results

### Model validation

**Figure 2:**
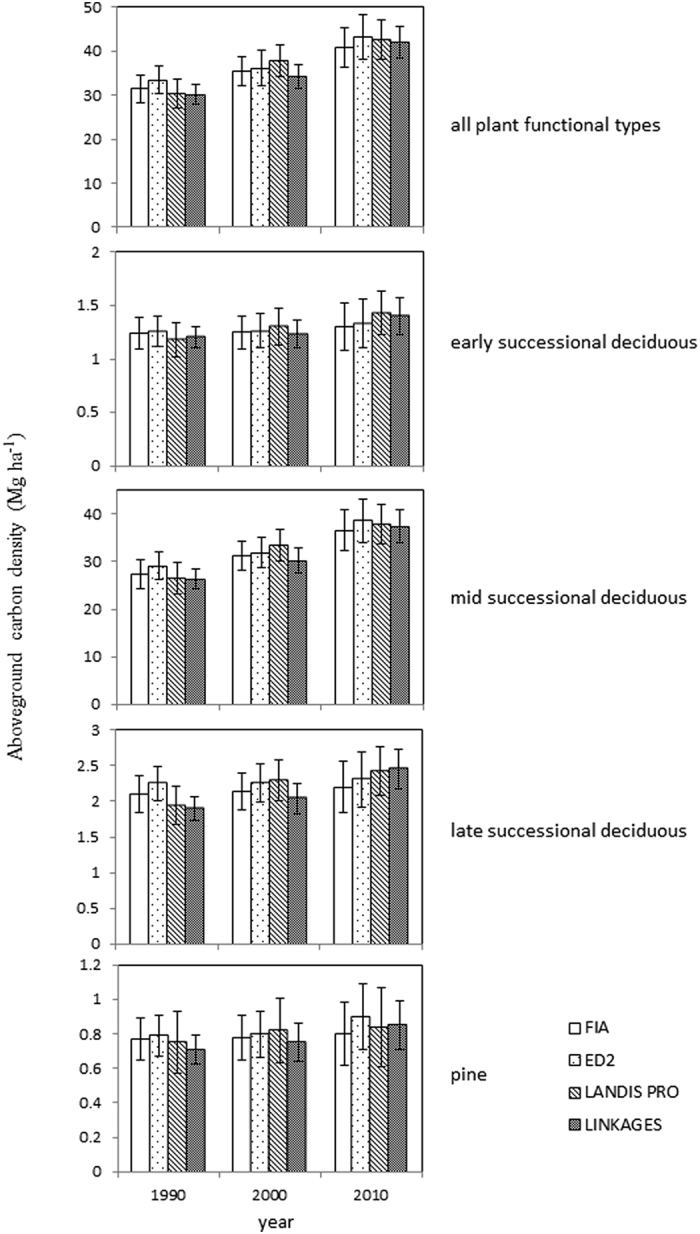
Comparisons of aboveground carbon densities of all plant functional types, early, mid, and late-successional deciduous trees, pines predicted by LINKAGES, ED2, and LANDIS PRO (all simulations were initialized with 1980 FIA data) with carbon density derived from FIA data of the Central Hardwood Forest in the United States. Error bars represent standard deviations among ecological subsections.

All three models showed reasonably good agreements with observed trends of a) increasing total aboveground carbon density and b) aboveground carbon densities of four plant functional types when compared with measured FIA plots from 1980 to 2010 (Fig. 2). Total aboveground carbon density increased from 27.2 to 40.9 Mg ha^−1^ from 1980 to 2010 according to the FIA data. Over the 30-year period, ED2 tended to overpredict total aboveground carbon density with a bias of 1.7 Mg ha^−1^, or 4.9%. LANDIS PRO tended to overpredict with a bias of 1.0 Mg ha^−1^, or 2.6%. LINKAGES tended to underpredict with a bias of −0.4 Mg ha^−1^, or −1.4%. Annual total aboveground carbon density changes from 1980 to 2010 for the FIA data and the ED2, LANDIS PRO, and LINKAGES predictions were 0.46, 0.54, 0.51, and 0.50 Mg ha^−1^ year^−1^, respectively. For early-successional deciduous trees, ED2 tended to overpredict aboveground carbon density over the 30-year period, and so did LINKAGES and LANDIS PRO. For mid-successional deciduous trees, ED2 tended to overpredict aboveground carbon density, LANDIS PRO tended to overpredict, and LINKAGES tended to underpredict. For late-successional deciduous trees, ED2 tended to overpredict aboveground carbon density, LANDIS PRO tended to overpredict, and LINKAGES tended to underpredict. and. For pines, ED2 tended to overpredict aboveground carbon density, LANDIS PRO tended to overpredict, and LINKAGES tended to underpredic (Fig. 2).

### Trajectories of carbon dynamics

**Figure 3:**
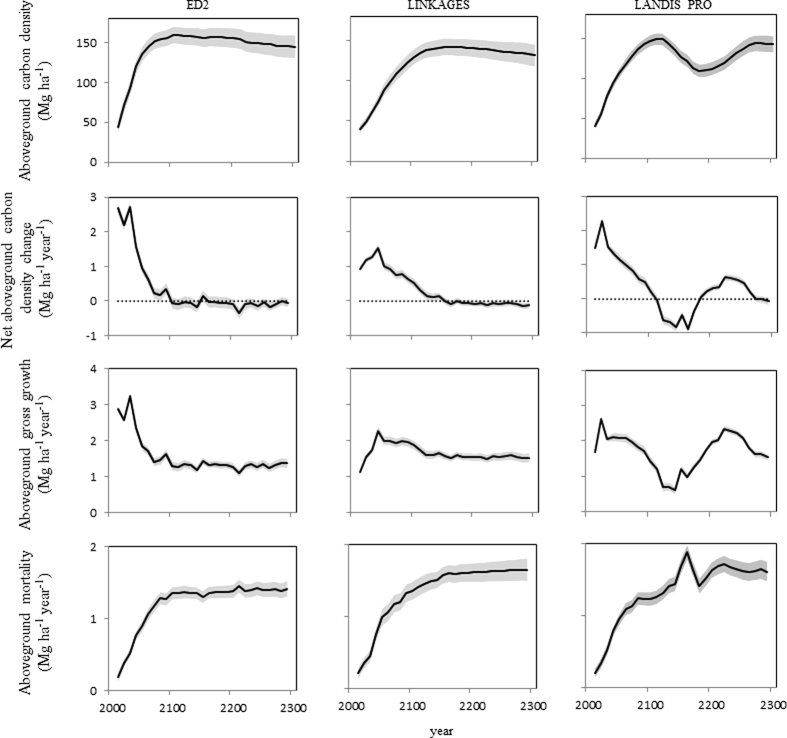
Aboveground carbon density, net aboveground carbon density change, aboveground gross growth, and aboveground mortalityfrom 2010 to 2300 for the Central Hardwood Forest in the United States predicted by LINKAGES, ED2, and LANDIS PRO. Gray ribbons represent standard deviations among ecological subsections.

Projected trajectories of carbon density differed among models. However, LINKAGES and ED showed similar trends: aboveground carbon density increased over time to a maximum followed by a slight decrease to the end of the simulation period in 2300 (Fig. 3). Despite similar overall trends, maximum aboveground carbon densities varied from 142.2 (LINKAGES) to 159.8 (ED2) Mg ha^−1^, and the time in which the maximum densities were reached also varied greatly from 2100 (ED2) to 2160 (LINKAGES) (Fig. 3). After maximum aboveground carbon densities were reached, the lowest aboveground carbon densities were observed in 2300 in both models. LANDIS PRO showed a different trend; aboveground carbon density increased to a peak of 149.6 Mg ha^−1^ in 2110, decreased to 109.9 Mg ha^−1^ in 2180, then increased to an asymptote of approximately 143 Mg ha^−1^ in 2260 (Fig. 3).

Net aboveground carbon density changes predicted by all models were positive from 2010 to 2090. Within this period, maximum annual net aboveground carbon density changes were 2.7, 2.3 and 1.5 Mg ha^−1^ year^−1^ for, ED2, LANDIS PRO, and LINKAGES, respectively. Annual net aboveground carbon density changes dropped below zero in 2100, 2110, and 2160 for ED2, LANDIS PRO, and LINKAGES, respectively. Thereafter, the annual net aboveground carbon density changes remained slightly negative most of the time for ED2 and LINKAGES. However, annual net aboveground carbon density change for LANDIS PRO showed a peak-dip-recovery pattern: it was positive from 2010 to 2100, negative from 2110 to 2170, and positive from 2180 to 2260 (Fig. 3). Trends of aboveground gross growth predicted by all three models were similar to those of net aboveground carbon density change. Average aboveground gross growths over the whole simulation period were 1.56, 1.69, and 1.67 Mg ha^−1^ year^−1^ for ED2, LANDIS PRO, and LINKAGES, respectively. Aboveground mortality predicted by ED2 showed an increasing trend from 2010 to 2100, and then plateaued around 1.38 Mg ha^−1^ year^−1^. Aboveground mortality predicted by LANDIS PRO increased from 2010 to 2140, followed by a peak of mortality (1.88 Mg ha^−1^ year^−1^in 2160), and decreased to 1.41 Mg ha^−1^ year^−1^ in 2180. The aboveground mortality increased again to 1.72 Mg ha^−1^ in 2220, and decreased slightly to 1.61 Mg ha^−1^ at the end of the simulation period (Fig. 3). Aboveground mortality predicted by LINKAGES showed an increasing trend from 2010 to 2180, and then stayed around 1.63 Mg ha^−1^ year^−1^.

**Figure 4:**
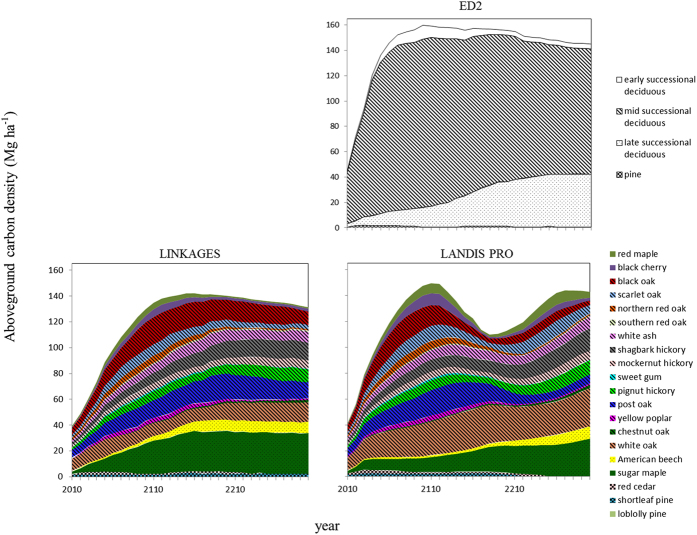
Carbon density by species (LINKAGES and LANDIS PRO) and by plant functional types (ED2, LINKAGES, and LANDIS PRO) from 2010 to 2300 for the Central Hardwood Forest in the United States. Species assigned for each plant functional type can be found in Table 1.

Both ED2 and LINKAGES predicted increases in aboveground carbon density of shade-intolerant species from 2010 to approximately early 22^nd^ century, followed by declines towards 2300. However, LANDIS PRO predicted a peak-dip-recovery pattern in aboveground carbon density of shade-intolerant species: it increased until 2110, declined until 2180, and then increased again. All models predicted that mid-successional species contained the largest portion of aboveground carbon throughout the simulation period. All models predicted increases in aboveground carbon density of shade-tolerant, late-successional species at the end of simulation period at which time they accounted for approximately 30% of the total aboveground carbon. Aboveground carbon density of pines remained low and relatively stable in all three models (Fig. 4).

## Discussion

Durations of the period in which the CHF was a carbon sink varied among the three models (Fig. 3); however, there was consensus that the period the CHF was a carbon sink would persist to at least 2100. The current average age of the CHF is approximately 70 years, and by 2100, the average age would be approximately 160 years. This duration of the carbon sink (approximately 170 years) is consistent with that proposed by Bormann and Likens^10^. It is also consistent with model predictions suggesting temperate forests in Massachusetts will remain as carbon sink until at least to 2060 due to forest regrowth^31^. However, our prediction for the duration of the carbon sink was much younger than 350 years e.g.^11^,^12^,^13^, and older than 80 years^9^ as reported in other temperate forests.

Average net aboveground carbon density changes predicted by ED2, LANDIS PRO, and LINKAGES from 2010 to 2300 were 0.35, 0.35, and 0.32 Mg ha^−1^ year^−1^, respectively. All values were lower than 0.51 Mg ha^−1^ year^−1^, which was derived from the 30-year (1980-2010) FIA data of younger forests. And a general decreasing trend of annual net aboveground carbon density change can be found in predictions of all three models. Such a decreasing trend is in accordance with the consensus that older forests typically have lower annual net aboveground carbon density change than younger forests^32^,^33^,^34^. Decreasing annual net aboveground carbon density change was likely predicted because gross growth (birth and subsequent growth) of aging forest gradually decreases to equilibrate with mortality^35^, and when gross growth equals to mortality the annual net aboveground carbon density change becomes zero.

Both simple and complex demographic approaches (ED2 and LINKAGES) predicted more stable aboveground carbon density trajectories, with slight decrease towards the end of simulations. These two models differ in the way they simulate birth but both simulate mortality resulting from slow growth and longevity. However, ED2 simulates effects of small-scale disturbances through density-independent mortality rates for different plant functional types, while LINKAGES does not simulate such disturbances. These results were consistent with studies suggesting that mortality plays a significant role in carbon dynamics of temperate forests e.g.^14^,^15^. The decline in aboveground carbon density predicted by LANDIS PRO was largely caused by both concentrated longevity-related mortality in comparatively short-lived, shade-intolerant species and decrease in aboveground gross growth (Fig. 3). These short-lived, shade-intolerant species included black oak, red maple, black cherry, scarlet oak, and northern red oak (Fig. 4), and their average longevities are 150–200 years. In the period between 2110 and 2180, most current cohorts of these species will reach their longevities, hence there is a peak in the predicted aboveground mortality (Fig. 4). On the other hand, in LANDIS PRO, regeneration success rates of these species were set to be low due to limited germination and competition for light in the presence of larger and older tree cohorts^36^. Such limited regeneration probabilities could contribute to the decline in aboveground gross growth due to limited carbon density increase contributed by younger cohorts. After a period of high, longevity-related mortality due to the clustered age-class distribution, forest gaps are expected to form and facilitate regeneration opportunities for the relatively short-lived and shade-intolerant species^37^. Due to a lack of long-term forest inventory data and the relatively young mean age of CHF, we can’t directly validate the predicted peak-dip-recovery pattern characteristic of the LANDIS PRO simulation. Such a pattern is supported, however, by relatively discontinuous yet concentrated (multi-peak) age distributions reported from several types of forests with much older mean ages that include oak forests (e.g., *Quercus mongolica* var. *grosseserrata*) in northern Hokkaido, Japan^38^, and coniferous forests (e.g., *Abies balsamea* and *Tsuga canadensis*) in the Great Lake region^39^.

After reaching a peak, aboveground carbon density predicted by ED2 and LINKAGES declined slowly until 2300 (Fig. 4). This pattern differed from that of LANDIS PRO mainly in that both simple and complex demographic approaches did not predict the peak-dip-recovery carbon dynamics of shade-intolerant species. This difference can be partly attributed to different model assumptions and formulations. Both ED2 and LINKAGES simulate mortality as a function of slow growth (small carbon/diameter increment or negative carbon increment) in the event of unfavorable environment conditions, e.g., excessively high temperature^16^,^19^,^40^. In contrast, competition-related mortality in LANDIS PRO is modeled directly as a function of stand density (i.e. self-thinning), rather than as a function of estimated tree growth rates^17^. Another difference among models is that regeneration events are generally more continuous in ED2 and LINKAGES than in LANDIS PRO. In ED2, regeneration of any plant functional type is not limited by environmental conditions or physiological attributes^40^. Regeneration events are more episodic in LINKAGES in that both physiological attributes and light conditions (e.g. inter-tree competitios for light) determine if a given species could regenerate in a plot^19^. A more continuous regeneration modelling process could, at least partially, offset both decreasing gross growth in aging forests and carbon density loss due to mortality of large, old trees. That would result in a smother curve describing aboveground carbon density over time. Another difference among models in regeneration estimation methodologies is that LANDIS PRO explicitly tracks the age of cohorts^17^, while the other two models track diameter^16^,^19^. All three models have built-in conversions to infer diameter of an individual tree or cohort from age (for LANDIS PRO which tracks age), or to infer age of an individual tree or cohort from individual/cohort diameter (for ED2 and LINKAGES that track tree diameter). However, ages in models that track diameter could be more likely distorted along the course of simulation, resulting in a forest more diverse in structure. For instance, drought could reduce diameter increment, on one hand, severe reduction in diameter increment could trigger mortality; on the other hand, if drought is not severe enough to kill tree, smaller diameter increment would lead to underestimated age. Regeneration and mortality in more diverse forests could be less concentrated than those in forests with concentrated age distribution, thus reducing the fluctuation magnitudes of forest carbon dynamics. The overall decreasing aboveground carbon densities predicted by ED2 and LINKAGES were attributed to declines in early- and mid-successional species, despite increases in late-successional species. And declines in mid-successional species were major contributions in total aboveground carbon density declines for both models (Fig. 4).

It should be noted that this study only focused on dynamics of carbon stored in live, aboveground biomass. However, carbon is sequestered also in slowly decomposing dead organic matter in other carbon pools (e.g., soil and deadwood). The soil carbon pool has been shown to be able to slowly accumulate carbon in old-growth forests^41^. A study found that soil in a mature temperate forest (regrowing from agricultural abandonment in the late 19th century) in Massachusetts is still accumulating carbon, which accounts for 5–15% of the total ecosystem carbon sink^42^. Such deadwood carbon pools have the potential to partly offset carbon losses from the live carbon pool as trees that are predicted to die leave the live carbon pool and enter the deadwood carbon pool. Through decomposition, carbon in deadwood will leave the forest ecosystem and return back to the atmosphere, but for large trees this process takes years. However, in this modeling study when trees are predicted die the carbon in those trees is immediately excluded from the aboveground carbon density and no longer accounted for, which could underestimate carbon sink strength of the forest ecosystem.

Our multi-model ensemble provided an approach that helped to achieve consensus on a general pattern in forest aboveground carbon dynamics, and to highlight possible causes for differences. Our results, however, should be interpreted within the context of key assumptions of forest demographic processes. We focused on a baseline scenario that considered the effects of forest regrowth on aboveground carbon dynamics and did not include climate change or disturbance. Under climate change scenarios, average aboveground carbon density was expected to be lower than those under current climate in the CHF. And patterns of aboveground carbon density change would likely remain largely the same (e.g., year in which peak of carbon density predicted by LANDIS PRO occurs will not change much) if only climate change is considered. Furthermore, there could be some long-term forest composition changes under climate change scenarios^43^. We also purposely did not simulate major disturbances such as harvest or fire, which can be more influential drivers of aboveground carbon dynamics in temperate and boreal forests than climate change alone e.g.^44^,^45^,^46^,^47^,^48^,^49^. Disturbances could decrease the magnitude of the carbon sink by preventing forest carbon density from reaching maximum capacity. Alternatively, disturbance could reduce the magnitude of a carbon source if it diversifies species composition, age, and size structure, because large, longevity-related mortality events like those illustrated with LANDIS PRO would be less likely. We also assumed that there was no land use change, and the total area of forest remained constant for the duration of all scenarios.

Although forest response to global change pressures, e.g., climate change, was not a focus of this study, there are insights into the role of demographic processes–specifically gross growth and mortality–on long-term aboveground carbon dynamics of temperate deciduous forests in the central United States. On one hand the simple, intermediate, and complex demographic approaches all agreed that the current CFH carbon sink would last at least to 2100. On the other hand, great uncertainties lie in the aboveground carbon dynamics beyond 2100 after the current carbon sink diminishes, and the uncertainties are related to different assumptions and formulations in gross growth and mortality estimation. Despite uncertainties about forest dynamics after 2100, the models all agree that following roughly a century serving as a massive carbon sink, the forests of the Central Hardwood region abruptly become carbon neutral or a carbon source. We suggest that future model design and development should attempt to improve simulating longevity-related mortality and regeneration of short-lived, shade-intolerant species in order to reduce uncertainties in long-term aboveground carbon dynamics of temperate deciduous forests. Future work should also examine how alternative management practices could alter projected patterns of carbon dynamics.

## Additional Information

**How to cite this article**: Jin, W. *et al*. Future forest aboveground carbon dynamics in the central United States: the importance of forest demographic processes. *Sci. Rep.* **7**, 41821; doi: 10.1038/srep41821 (2017).

**Publisher's note:** Springer Nature remains neutral with regard to jurisdictional claims in published maps and institutional affiliations.

## Supplementary Material

Supplementary Information

## Figures and Tables

**Table 1:** 20 tree species and the corresponding 4 plant functional types included in this study.

Plant functional type	Common name	Scientific name
Early-successional deciduous	red maple	*Acer rubrum* L.
	black cherry	*Prunus serotine* Ehrh.
Mid-successional deciduous	pignut hickory	*Carya glabra* Miller
	shagbark hickory	*Carya ovata* (Mill.) K. Koch
	mockernut hickory	*Carya tomentosa* Sarg.
	white ash	*Fraxinus americana* L.
	sweet gum	*Liquidambar styraciflua* L.
	yellow poplar	*Liriodendron tulipifera* L.
	white oak	*Quercus alba* L.
	scarlet oak	*Quercus coccinea* Muenchh.
	southern red oak	*Quercus falcata* Michx.
	chestnut oak	*Quercus prinus* L.
	northern red oak	*Quercus rubra* L.
	post oak	*Quercus stellata* Wangenh.
	black oak	*Quercus velutina* Lam.
Late-successional deciduous	sugar maple	*Acer saccharum* Marshall
	American beech	*Fagus grandifolia* Ehrh.
Pine	red cedar	*Juniperus virginiana* L.
	shortleaf pine	*Pinus echinata* Mill.
	loblolly pine	*Pinus taeda* L.
