# Supplementary material for: Future forest aboveground carbon dynamics in the central United States: the importance of forest demographic processes
Source: Sci Rep. 2017 Feb 6;7:41821. doi: 10.1038/srep41821 (PMC5292963; doi:10.1038/srep41821)
Supplement: Supplementary Information [file srep41821-s1.docx]

**Model description and parameterization**

***ED2***

ED2 is a detailed physiological ecosystem model that is multi-scale and highly mechanistic [^16^](#_ENREF_1). It incorporates phenomena from fine scale plant physiological response to hourly environmental forcing, weekly and seasonal changes in soil hydrology, and long-term changes in forest composition and carbon storage. Aboveground and belowground ecosystem structure and fluxes of water and carbon between the ecosystem and the atmosphere are simulated [^40^](#_ENREF_2). ED2 does not simulate individual species but simplifies species into several plant functional types, for which we used early-, mid-, and late-successional temperate deciduous trees and southern pines in this study. A system of partial differential equations is used to approximate the mean behavior of a corresponding stochastic gap model to simulate population-level heterogeneity represented by different plant cohorts, which are groups of plants with the same plant functional type and similar size and disturbance history. All plant functional types are given the same rate of birth and minimum size, regardless of their physiological differences. Mortality is related to functional-type-specific longevity and prolonged low or negative carbon balance [^16^](#_ENREF_1), density-independent mortality rate were also assigned to each plant functional type ^40^. ED2 subdivides the landscape into grid cells ranging from 10^-1^ to 10^2^ km, and it can thus simulate ecosystem dynamics varying from grid cell scale to regional scale.

We gridded the landscape into 1° cells for ED2. Stand ages of FIA plots which fall within a given ED2 cell was classified into different age classes with a 10-year age increment, each stand age class was assigned to a patch in an ED2 cell. Cohorts in each patch were initialized with plant functional type, stem density and DBH from FIA plots. We used climate parameters at the centroid of each grid cell and calculated hourly air temperature, air pressure, specific humidity, wind speed, precipitation, and incoming longwave and shortwave radiation by linearly interpolating the 6-hour NCEP/NCAR reanalysis data set from 1980 to 2009. We obtained soil textural classes and depth of mineral soil from the Web Soil Survey and used average soil parameters for each cell. We adapted physiological parameters from [^30^](#_ENREF_5).

***LANDIS PRO7.0***

LANDIS PRO 7.0 is a spatially explicit, grid cell based forest landscape model operating at large spatial scales (10^6^-10^8^ ha) and long temporal scales (10^2^-10^3^ years) with flexible spatial (30-500m) and temporal resolutions (1-10 years). One unique feature of this model is that it can simulate individual-species demography (birth, growth, and mortality) and stand dynamics within each stand (grid cell) while simulating landscape processes (e.g., dispersal, harvest) across the region ^17, 18^. The model tracks number of trees and DBH for each species age cohort within each grid cell. Density and age are directly derived from forest inventory data and DBH for each age cohort is subsequently updated according to prescribed age-DBH relationships, which vary by land types [^17^](#_ENREF_6). Aboveground biomass of individual species is calculated from DBH and density using species specific allometric models and carbon is estimated as half the amount of biomass. Birth is determined by species-specific traits, such as shade-tolerant class, and responses to environmental conditions. Besides random background mortality, mortality is also a function of longevity, competition, and disturbances. LANDIS PRO regulates stand development using growing space occupied and simulates self-thinning once stands exceed the maximum growing space capacity.

We gridded the landscape into 270 m cells for LANDIS PRO. We used Landscape Builder for LANDIS PRO to assign initial vegetation conditions to each pixel by randomly selecting from among homogenous groups of FIA plots that we grouped by region, land cover, and land form. Species life history attributes, e.g., longevity, were adopted from previous studies [e.g., ^17^](#_ENREF_6). LANDIS PRO does not require climate and soil parameters, however, we derived species establishment probability (SEP) and maximum growing space by landtype from the Linkages model described above to reflect differences across a gradient of environmental conditions ^17^.

***LINKAGES*** *v2.2*

LINKAGES v2.2 is a hybrid empirical-physiological model that simulates long-term forest dynamics and ecosystem carbon and nitrogen cycles using plot-level data [^19^](#_ENREF_11). It assumes interactions between tree demographic, biogeochemical and microbial processes can explain much of the variation in forest dynamics and ecosystem carbon and nitrogen cycling. LINKAGES is a plot-scale model with a daily time step. It simulates birth, growth, and mortality of individual trees that have a diameter at breast height (DBH) larger than 1.4 cm. This model does not simulate forest management or natural disturbances such as fire and insect outbreak; however, it can simulate drought based on input climate data. Tree growth and reproduction are controlled by estimated availability of light, soil water availability, soil nitrogen, and growing degree days. Birth is controlled by number of seeds, available light at the forest floor, and species’ shade tolerance. Mortality is probabilistic and is affected by unfavorable climate conditions, such as prolonged droughts, slow growth, and species’ longevity. The model’s state variable DBH and carbon density is estimated from it using one allometric model for all species.

We assigned FIA plots to ecological subsections and used climate parameters from the centroid of each ecological subsection and average soil parameters for the subsection. Species-specific parameters were derived from species’ life history attributes (e.g., longevity, maximum and minimum growing degree days) ^30^. We obtained daily air temperature, precipitation, solar radiation and wind speed for 1980 to 2009 from the NCEP/NCAR reanalysis data set. We obtained estimates of wilting point; water-holding capacity; and average percentage of clay, sand, and rock in the uppermost 1 meter soil from the Web Soil Survey. We used a modified version of LINKAGES developed by Dijak et al. (under review) in which diameter growth increment models were replaced. We determined growth coefficients by fitting models to the 90th percentile of tree diameters for trees at ages 30, 60, 90 and 120 years based on FIA site index tree data.
